# Maternal Smartphone Use and Regulatory Disorders of the Infant: A Post Hoc Analysis of the SKKIPPI Cohort Study

**DOI:** 10.3390/children13091248

**Published:** 2026-09-15

**Authors:** Anne Berghöfer, Annika Luise Wrenger, Julia Fricke, Marie Bolster, Lars Kuchinke, Christiane Ludwig-Körner, Franziska Schlensog-Schuster, Thomas Reinhold, Stephanie Roll, Thomas Keil

**Affiliations:** 1 Institute of Social Medicine, Epidemiology and Health Economics, Charité University Medical Center Berlin, Corporate Member of Freie Universität Berlin and Humboldt-Universität zu Berlin, 10117 Berlin, Germany; annika.wrenger@charite.de (A.L.W.); marie.bolster@charite.de (M.B.); thomas.reinhold@charite.de (T.R.); stephanie.roll@med.uni-rostock.de (S.R.); thomas.keil@charite.de (T.K.); 2 Unit for Municipal Health Strategies for the City of Freiburg and the District of Breisgau-Hochschwarzwald, 79104 Freiburg, Germany; julia.fricke@lkbh.de; 3 International Psychoanalytic University, 10555 Berlin, Germany; lars.kuchinke@ipu-berlin.de (L.K.); christiane.ludwig-koerner@ipu-berlin.de (C.L.-K.); 4 University Hospital of Child and Adolescent Psychiatry and Psychotherapy, University of Bern, 3000 Bern, Switzerland; franziska.schlensogschuster@upd.ch; 5 Institute for Complementary and Integrative Medicine, University Hospital Zurich and University of Zurich, 8091 Zurich, Switzerland; 6 Institute for Biostatistics and Informatics in Medicine and Ageing Research, Universitätsmedizin Rostock, 18057 Rostock, Germany; 7 Institute for Clinical Epidemiology and Biometry, University of Würzburg, 97070 Würzburg, Germany; 8 State Institute of Health I, Bavarian Health and Food Safety Authority, 91058 Erlangen, Germany

**Keywords:** the questionnaire for crying, feeding and sleeping, M.I.N.I., mother–child interaction, regulatory disorder, smartphone use

## Abstract

**Highlights:**

**What are the main findings?**

- The parent’s use of a smartphone whilst interacting with his/her baby does not appear to be directly linked to regulatory difficulties in the child.
- Rather, it seems to be meaningful when the parent becomes stressed during smartphone use if the child seeks attention.

**What are the implications of the main findings?**

- The use of electronic devices in the baby’s presence should not be demonized out of hand.
- However, parents should be advised that a stressed or dismissive reaction towards the baby when it seeks attention may be linked to regulatory difficulties.

**Abstract:**

Background/Objectives: Infant regulatory disorders (RDs) describe the infants’ difficulties in regulating their physiological and emotional states, manifesting in problems with sleeping, feeding, or excessive crying. A post hoc analysis of data from the German SKKIPPI cohort study investigated the association of maternal smartphone use and the occurrence of RDs. Methods: In a randomly drawn sample from three local population registers, a self-administered online questionnaire was used to collect basic sociodemographic data, smartphone use behavior and the mother’s subjective perception of the infant’s RD. In case of psychosocial burden of the mother, a structured psychiatric-diagnostic interview (M.I.N.I.) was conducted and the Questionnaire for Crying, Feeding and Sleeping (SFS) was completed. Results: Of the 5954 parent–child dyads completing the online-questionnaire, 3.7% reported frequent smartphone use while interacting with the child, compared with 5.8% of the 972 mother–child dyads in the psychiatric interview. Among frequent smartphone users, 61.5% indicated RD compared to 41.6% of the infrequent users (*p* = 0.005). After adjustment for confounding by psychiatric disorder, educational level, single parent status, and age, perceived stress when interrupted during smartphone use was consistently associated with less favorable outcomes (OR of feeding easy 0.56, 95% CI 0.42; 0.75; OR of falling asleep easily 0.85, 95% CI 0.73; 0.99; OR of excessive crying 1.37, 95% CI 1.04; 1.81). RD based on the SFS was associated with elevated stress during smartphone use (OR 1.87, 95% CI 1.35; 2.58). Conclusions: Maternal stress when smartphone use is interrupted is more strongly associated with early regulatory difficulties than frequency of smartphone use alone, indicating the importance of also considering emotional and contextual factors in the analysis of technology use during parent–child interactions.

## 1. Introduction

### 1.1. Early Childhood Regulatory Disorders

Early childhood regulatory disorders describe a child’s developmental challenges in regulating behavior and physiological, sensory, motor, attention-related, or emotional processes. These difficulties affect both the child’s everyday adaptability and the interaction patterns between parent and child [1,2]. As infants and young children are still at the beginning of their physiological development, co-regulatory support from their caregivers, who are sensitive to the individual needs of their children and accompany them accordingly in the adaptation and development process, is indispensable [3,4]. Affective mirroring, in which the caregiver perceives the baby’s needs and emotions and responds appropriately in another sensory modality, is a prerequisite for the development of mentalization skills. In this way, they help the infant to regain a balanced state after irritation, agitation and/or dysregulation (e.g., crying). It can be assumed that adverse or unemotional experiences in the caregivers’ past lead to a reduced or imbalanced emotional exchange with their children in the context of transgenerational transmission [5].

This illustrates that regulatory disorders can arise from an imbalance in parental co-regulation processes and that child development and its potential problems should therefore always be considered in the context of the family environment [4,6]. Mostly, the described problems with affect, emotion and behavioral regulation manifest themselves in the form of excessive crying, sleep and feeding disorders. In addition, parents are emotionally burdened and overwhelmed by this condition, and dysfunctional interaction with the infant can develop, which can lead to the symptom triad of early childhood regulatory disorders. Around 20–25% of healthy children exhibit such symptoms in the first three years of life [4,7,8]. Various potential risk factors have already been investigated in earlier studies, and an association with psychosocial stress factors in the family has been established. Hiermann et al. reported from a counseling center that 23% of families had multiple stress factors, with 18% of mothers of affected children having symptoms of depression [7]. At the same time, there is evidence that regulatory disorders in early childhood are linked to behavioral problems in later childhood [9]. In this respect, paying attention to regulatory disorders and their family context is of great importance for prevention.

### 1.2. Effects of Parental “Technoference”

The ever-increasing use of digital devices in everyday life, especially smartphones, is fundamentally changing interpersonal communication. Depending on the family context, even very young children encounter media devices. In particular, an increase in the amount of time a mother spends using media is linked to greater media use among young children as well [10]. Particularly in the context of vulnerable situations such as the interaction between mother and infant, this raises the question of potentially negative effects on early childhood bonding.

The concept of ‘technology interference’ or ‘technoference’ describes the disruption, interruption or intrusion of technological devices into the personal communication and interactions of couples or families in everyday life [11,12,13]. Previous studies suggest that parents with younger children are more likely to use their smartphones to distance themselves from their children [13,14], especially when they find their role as parents frustrating or isolating [13,15]. There are also many other reasons why parents, especially mothers, use their smartphones while interacting with their babies: identifying with influencers to compensate for their own insecurity in their role as mothers or low self-esteem. Mothers may also seek stimulation and participation because they fear missing out on life or want to suppress depressive, anxious, or other negative feelings.

Smartphone use in face-to-face interactions can be associated with changes in parental responsiveness [16], and parents are less sensitive to their children’s attention needs, both verbally and non-verbally [14]. A recent systematic review and meta-analysis confirmed parents’ use of technology in their child’s presence being negatively associated with cognitive and psychosocial outcomes among young children [17].

Especially in infants and young children, who depend on the sensitive responsive behavior of their caregivers to develop secure attachment patterns, such disturbances can have a negative impact on the interaction processes between parent and child as well as on the child’s ability to self-regulate [16,18]. In two independent and similarly structured randomized controlled trials (RCTs), the classic still face paradigm was modified and applied comparatively to further characterize the effects of parental smartphone use, by replacing the still face phase with a phase of non-responsive smartphone use [19,20]. Since this classic experiment suggests the likelihood of negative consequences for a child’s social and emotional development, a possible transferability to smartphone use was discussed [19]. In addition, it was found in one of the experiments that frequent smartphone use by the caregiver in everyday life was associated with reduced positive affect, exploratory behavior and poorer recovery in the experiment [19]. In the second experiment, an increased protest reaction and reduced positive affect of the children was also observed during the smartphone use phase, suggesting comparability with the still-face effect [20]. Further modifications of this experimental approach confirmed the results [21]. In a systematic review focusing on the age group of zero to three-year-old children, reduced responsiveness and sensitivity on the part of parents was also identified, but the negative effects on children’s regulation and affect could only be identified during active smartphone use, but not beyond. The results on possible later implications were inconsistent, but an association of technoference with impaired child learning is discussed [22]. A study focusing on the child’s physiological response showed that the infant’s heart rate increased as a sign of agitation when play with the mother was interrupted by smartphone use [23]. Ultimately, however, there are indications that smartphone use during interaction with the child is not necessarily problematic, but rather that it is more relevant to what extent the mother remains sensitive to the child’s needs [24,25].

### 1.3. Aim of This Study

Using data from the epidemiological cohort study of the SKKIPPI project [26,27,28], the aim was to investigate whether there is an association between frequent smartphone use by the mother and the increased occurrence of early childhood regulatory disorders. Furthermore, the effect of potential confounding factors on the association was to be analyzed.

## 2. Materials and Methods

### 2.1. Study Participants and Study Design

In the epidemiological cohort study conducted as part of the SKKIPPI project, a random sample was randomly drawn from the three residents’ registration offices in the cities of Berlin, Leipzig and Flensburg. The sample included around 30,000 families with children up to the age of 12 months who were contacted and invited to participate. Recruitment took place between March 2019 and July 2020. With a response rate of 20%, a total of around 6000 parent–child dyads were included in the study. Participants had to be of legal age, biological or adoptive parents of the newborn and give written consent to participate in the study. In an initial screening step, the socio-demographic parameters and psychosocial burdens of the young parents were assessed. If psychosocial stress was present, the psychiatric-diagnostic telephone interview M.I.N.I. was conducted in a second screening step, and the previous use of health services and psychosocial support services were recorded. In a follow-up examination after 6 months, the psychiatric-diagnostic telephone interview M.I.N.I. was repeated, and utilization was again surveyed.

Two subsets of data were relevant to this post hoc analysis:

- Subset one consisted of those mothers and fathers who participated in the first online screening step (n = 5954) [26].
- Subset two consisted of those mothers (no fathers), who exhibited increased psychosocial distress based on the result of the first screening step [26] and underwent a diagnostic psychiatric interview and completed the standardized Questionnaire for Crying, Feeding and Sleeping (SFS) [28] (n = 972).

Detailed information on the multi-component project SKKIPPI (“Evaluation der Eltern-Säugling-KleinKInd-Psychotherapie mittels Prävalenz-und Interventionsstudien/Evaluation of parent-infant psychotherapy using prevalence and intervention studies”), a health services research project funded by the German Health Care Innovation Fund [29], was published previously [26,27,28,30,31,32,33]. Here, we shall present only a summary of the main SKKIPPI findings. An analysis of the first screening step data of SKKIPPI revealed that a substantial proportion of parents are burdened by psychosocial problems such as professional problems and a lack of social support and suffer from mental health problems in the first years after the birth of their children. Early preventive and low-threshold support measures should be available in the health care and social services system. Appropriate low-threshold questionnaires should be further developed for routine use in health care [26]. An additional examination of mothers with mental health burden in a second study step showed 5% of the mothers having at least one psychiatric disorder, with nearly half of them still affected at the 6-month follow up [28]. In a health economic analysis determinants of health care and early support services utilization were assessed. Most participants utilized some kind of early support services. Mothers with a noticeable mental health or psychosocial burden and their children caused total costs of €10,849 in the postnatal period from a payer’s perspective compared to €9136 for mothers without this burden [32]. As part of the SKKIPPI intervention studies, a focus-based brief parent–infant psychotherapy (PIP) was developed to support parents to promote a healthy parent–child relationship and child development. Compared to treatment as usual, PIP was not superior in enhancing maternal sensitivity, child symptoms of regulatory disorder or attachment security, but PIP seemed to reduce mothers’ symptoms of mental health problems [33].

### 2.2. Data Collection and Instruments

#### 2.2.1. Data Subset One

In the first screening stage of the SKKIPPI cohort study, data were collected using a questionnaire developed specifically for the study and addressing parents in the postpartum period [26]. It covers four major domains of stress: parent-related stress, family-related stress, parent–child interaction and child-related stress. It collects detailed data, including sociodemographic information, perinatal factors (e.g., planned/unplanned pregnancy, complications), parental stressors (e.g., relationship issues, financial or professional problems, past childhood trauma), and mental health problems in both parent and child. The screening tool was developed by experts, piloted, and iteratively refined. A scoring system was applied, with each of the 40 questions scored between 0 and 5. A score of 10 points or more indicated elevated psychosocial burden. The study is described in detail elsewhere [26,27].

For the present analysis, we selected the parameters that fundamentally characterize the socio-demographic properties of the study population and that could be possible confounders in the association between smartphone use and child regulatory disorders. Parameters age (in years), single parent status, receipt of state support and information on whether this is the first-born child were considered.

In addition, we recorded the respondents’ level of education and categorized it using the International Standard Classification of Education (ISCED), divided into the levels ISCED l (corresponding to primary education), ISCED Il (corresponding to completion of secondary level l), and ISCED III (corresponding to completion of secondary level Il) and other [34]. For the test of a possible association of the level of education with the outcome, a new dichotomous variable was calculated due to the low number of cases in the ISCED l group, whereby ISCED l and Il were classified as low to medium level of education and ISCED III as high level of education. The “other” group was not included here, as no statement could be made about the classification.

Furthermore, data on smartphone use behavior while feeding or playing with the infant were used. For this purpose, the data were converted into a dichotomous variable, whereby the answer option “frequently” was classified as positive exposure, while the options “sometimes”, “rarely”, and “basically not” were recoded as negative exposure. Further information from the screening questionnaire was used to determine whether the parent feels stressed when the infant demands attention while the parent is busy with the mobile device.

#### 2.2.2. Data Subset Two

Mothers who achieved a score of 10 or more in the first screening stage were examined in a second screening stage in a psychiatric-diagnostic telephone interview. For this purpose, the Mini-International Neuropsychiatric Interview (M.I.N.I.) was used, which enables valid diagnoses of mental disorders to be made according to DSM-V and ICD-10 in a structured manner using standardized questions [35]. The conduct of this part of the study is described in detail elsewhere [28]. For the post hoc analysis of subset two, the data of the individually available diagnoses of mental disorders were recoded into a dichotomous variable (0 = no diagnosis, 1 = at least one diagnosis), whereby isolated suicidality, which was also recorded, was not considered, as it represents a symptom.

The Questionnaire for Crying, Feeding and Sleeping (SFS) was used in the second screening step of the SKKIPPI cohort study to quantify and assess the infants’ regulatory problems. The SFS consists of 49 items, most of which are collected for the complexes “crying, whining, sleeping”, “feeding”, and “co-regulation” using four-level frequency scales. From these results, a partial score can be determined for each complex, from which a total score is calculated. The total score can adopt values from a minimum of one to a maximum of four, whereby the explicit diagnosis of regulatory problems is defined by a total value of ≥1.85 [36]. In the present analysis, both the mean values of the scores and a dichotomous variable that divides the data at the cut-off value of 1.85 into a group with and without a regulatory disorder (SFS < 1.85 = 0, SFS ≥ 1.85 = 1) were analyzed. Table 1 provides an overview of the variables used in the subsets.

Screening stage two of the SKKIPPI cohort study no longer included the fathers. Specific differences in postnatal psychosocial problems between mothers and fathers were reported elsewhere [37].

### 2.3. Statistical Analysis

The basis for the statistical analysis was subsets one (n = 5954) and two (n = 972) of participants from the SKKIPPI cohort study. The statistical analysis was performed with IBM SPSS Statistics Version 30.0.0.0 (Computer Software. Armonk, NY, USA). Variables in Table 1 were used to describe the populations using descriptive statistics.

To investigate the association between caregiver smartphone use and child regulatory disorder, the two strata, “smartphone use frequently” and “smartphone use rarely”, were compared using a robust Mann–Whitney U test for metric variables and a chi-square test for categorical variables. Further strata, such as maternal mental illness (from subset two), educational level, and perceived stress during smartphone use were analyzed exploratively.

Binary logistic regression was used to calculate odds ratios, including 95% confidence intervals for the occurrence of parent-reported feeding, crying and sleeping problems of the child from data subset one and SFS diagnosis of child regulatory disorder based on the SFS scale values from data subset two. Finally, a generalized linear model was calculated to determine the association between smartphone use and SFS subscores and SFS total score. All analyses included potential confounders. The variables maternal mental illness, education level, single parent status, age, stress during smartphone use, and the main variable frequency of smartphone use while interacting with the child were included as fixed covariates. No adjustment was made for multiple testing; missing values were not replaced. The results are interpreted purely exploratively.

## 3. Results

### 3.1. Study Population

**Figure 1:**
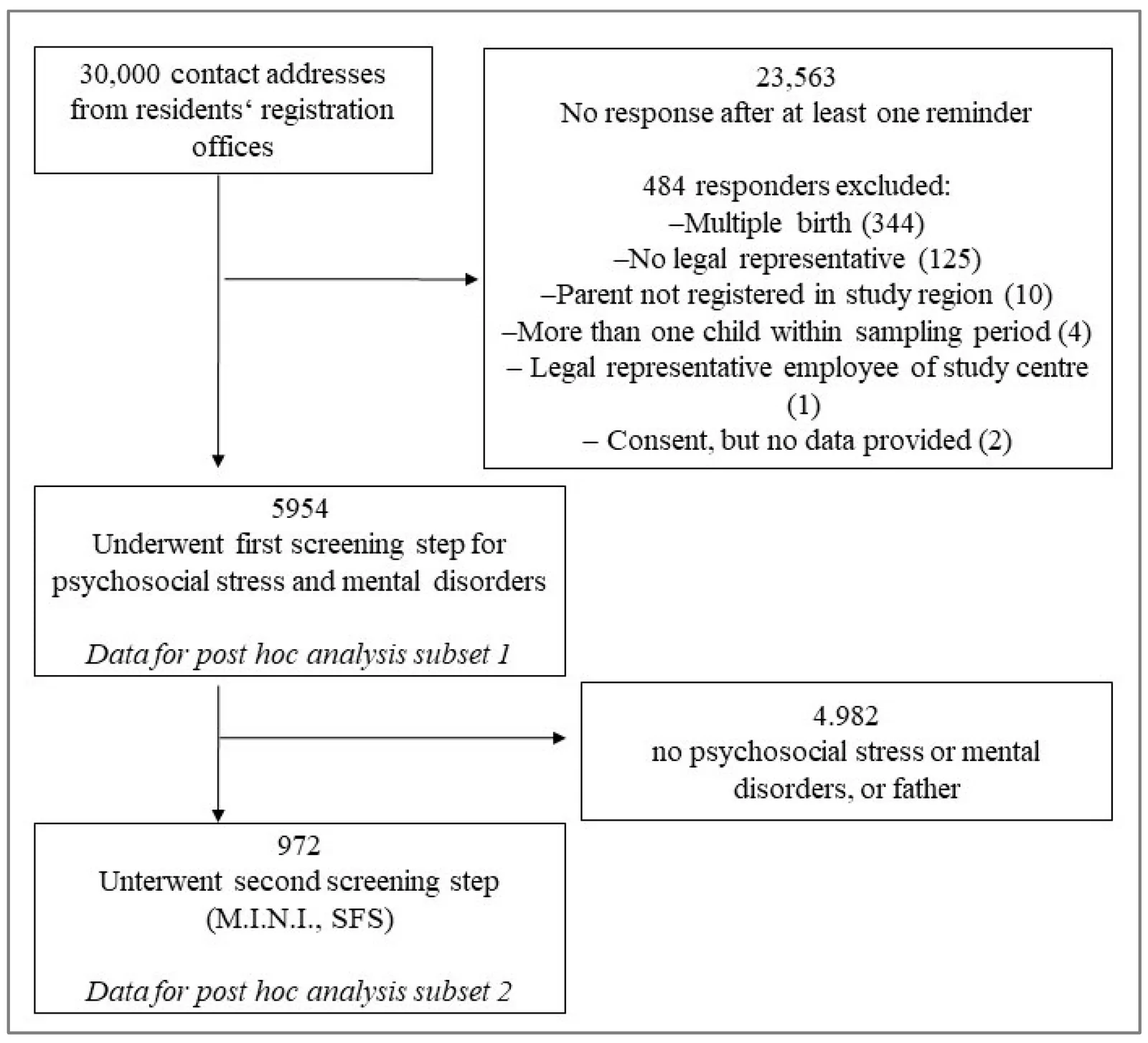
Flow chart of recruitment of study participants in the SKKIPPI cohort study [26] and selection of data subsets for post hoc analyses one and two.

The two subsets for the post hoc analyses consist of 5954 parent–child dyads and 972 mother–child dyads, respectively (Figure 1).

Of all parents who completed the first screening stage of the cohort study, 1697 (28.5%) reported using the smartphone often or sometimes while interacting with their child. Table 2 presents sociodemographic and anamnestic data from a sample of 5954 parent–child dyads, stratified by the parent’s smartphone use.

The average age of participants was slightly lower in the frequent use group compared to the infrequent group. All other parameters, such as educational levels, proportion of single parents, receipt of state benefits or experience of mental health problems did not show relevant differences between both groups. A notable difference emerged in reported stress during smartphone use while the child demands attention: 31.7% of frequent users reported stress compared to only 15.2% of infrequent users. Feeding difficulties were reported approximately twice as often among frequent users (9.2%) than among infrequent users (4.3%), but no relevant group differences were found for child’s falling asleep easily or excessive crying (Table 2).

Of the mothers in subset two, 56 (5.8%) stated that they frequently used their smartphone while feeding or playing with their child, while 916 (94.2%) stated that they only did so sometimes, rarely or not at all. The two groups differed slightly in terms of socio-demographic variables. The proportion of mothers with a high level of education (ISCED III) in the group with frequent smartphone use was 9.6 percentage points higher than in the group with infrequent smartphone use (Table 3).

The analysis of the SFS values based on data subset two revealed that slightly higher mean values were observed in the subscores “crying, whining, sleeping”, “co-regulation”, and “feeding”, as well as in the sum score in the children of mothers with frequent smartphone use compared to the children of mothers with low smartphone use. A sum score of ≥1.85 is defined as the threshold for regulatory problems. Based on this definition, children of mothers with frequent smartphone use had a relatively more frequent diagnosis of a regulatory disorder than children of mothers with infrequent smartphone use in the validation study of the SFS [36].

### 3.2. Exploring Confounding Factors

Due to the differences in smartphone use behavior in the subgroups with different levels of education and the association between maternal mental illness and the occurrence of regulatory disorders in the infant identified in previous studies [7], these characteristics were suspected to be potential factors influencing the association between exposure and outcome. Because frequent smartphone users were younger than infrequent users, age was also included as a confounder. Furthermore, the level of stress experienced during smartphone use when the child demanded attention was also included, because the user groups showed relevant differences in this regard. Since single parent status can be associated with subjective stress, this variable was also included as a confounder.

Using data subset two, strata were compared exploratively. In order to investigate the association between the level of education and the occurrence of regulatory disorders, the mothers were stratified into a group with a low level of education (ISCED I and II, n = 125) and a group with a high level of education (ISCED III, n = 699), excluding the “other” group. When analyzing the partial and total scores of the SFS questionnaire, a relevant difference was only found for the co-regulation subscore 2.42 (SD 0.6) for the children of mothers with a high level of education versus 2.25 (SD 0.6) for the children of mothers with a low level of education (*p* = 0.007). When using the diagnostic criterion (SFS sum score ≥ 1.85), there was no difference between the groups (n = 53 (42.4%) vs. 298 (42.6%)).

To investigate the relevance of a mental illness in the mother, the mothers were stratified into a group with 603 mothers without a mental illness and a group with 231 mothers who met the criteria for a diagnosis of at least one mental illness according to M.I.N.I. (n = 834 data sets in analysis). For the total score of the SFS and the subscores “crying, whining, sleeping” and “feeding”, significant but not relevant differences were found between the children of mothers with mental illness and the children of mothers without mental illness (1.86 (SD 0.3) vs. 1.83 (SD 0.3), *p* = 0.040; 1.80 (SD 0.4) vs. 1.73 (SD 0.4), *p* = 0.014; 1.49 (SD0.3) vs. 1.43 (SD 0.4), *p* = 0.037). No differences were found for the “co-regulation” subscore (2.40 (SD 0.6) vs. 2.39 (SD 0.6), *p* = 0.900). While 248 (41.1%) of infants whose mothers without psychiatric diagnosis had a diagnosis of regulatory disorder (SFS ≥ 1.85), this was the case for 109 (47.2%) infants of mothers with at least one psychiatric diagnosis (*p* = 0.114). Mothers with at least one psychiatric diagnosis reported relevantly higher stress when their child demanded attention while using a mobile device than mothers without a psychiatric diagnosis (83 of 267 (31.1%) vs. 157 of 705 (22.3%), *p* = 0.004).

### 3.3. Association Between Parents’ Smartphone Use and Parent-Reported Child Regulatory Disorder

To estimate the association between caregivers’ smartphone use and the infants’ regulation disorder, multivariate models were fit.

A binary logistic regression including all potential confounding factors was calculated for the dichotomous variables ease of feeding, ease of falling asleep and excessive crying from data subset one. Regarding the parent-reported ease of child feeding, both parental smartphone use and parental stress when disturbed during smartphone use were significantly associated with lower odds of finding child feeding easy. Specifically, higher smartphone use and increased stress when disturbed during use were linked to a reduced likelihood of perceiving child feeding as easy. In contrast, factors such as being a single parent, parental educational level, the experience of a psychiatric disorder, and parental age did not show associations with this outcome (Table 4).

In terms of parent-reported ease of falling asleep, parental stress when disturbed during smartphone use again emerged as a significant factor, being associated with lower odds of falling asleep easily. Similarly, being a single parent and the presence of a psychiatric disorder in the parent were also linked to decreased ease of falling asleep. Interestingly, parental age was positively associated with falling asleep easily, indicating a slight increase in odds with increasing age. However, the effect sizes for these factors were very small and therefore of little clinical relevance. It is worth noting that general smartphone use and parental education level did not reach significance in this context (Table 4).

For the outcome of parent-reported excessive crying, parental stress when disturbed during smartphone use was significantly associated with higher odds of excessive crying, although the effect size was very small. Being a single parent was also linked to increased likelihood of excessive crying. Conversely, higher parental age was associated with lower odds of excessive crying with, however, a negligible effect size. Other variables, including general smartphone use, education level, and experience of psychiatric disorders, did not show relevance (Table 4).

Overall, the results indicate that, in addition to frequent parental smartphone use, parental stress when disturbed or interrupted during smartphone use because the child needs attention is consistently associated with less favorable outcomes across all three domains. This finding highlights that it is not just the frequency of smartphone use, but also the stress felt when one is interrupted while using it, that may be an important factor in understanding child well-being in the context of smartphone use.

As part of a sensitivity analysis, we included all sociodemographic variables; in addition to the variables reported in Table 4, we also included the variable ‘receipt of social security benefits’ and the variable ‘index child was the parents’ first child’. The strength of the association between the individual factors did not change; however, the variable ‘index child was the parents’ first child’ also proved to be associated with the child’s regulatory difficulties as reported by the parents (parent-reported feeding difficulties: first child OR 0.68 (95 CI 0.51; 0.89), *p* = 0.005; parent-reported excessive crying of child: benefits receipt OR 1.62 (95 CI 1.20; 2.19), *p* = 0.002; parent-reported sleeping difficulties of child: first child OR 0.66 (95 CI 0.58; 0.75), *p* ≤ 0.001).

In a second sensitivity analysis, we re-examined the association of parental smartphone use, perceived stress during smartphone use when the child demands attention, parents’ demographic characteristics, and self-reported lifetime psychiatric experience (n = 5954 from data subset one) with parent-reported regulation disorders of the child using the data from subset one while excluding those mothers whose data are included in subset two. In this way, we generated two completely independent subsets one and two. This gave us a sample in subset one that was completely independent of data subset two. This analysis confirmed that perceived stress during smartphone use when the child demands attention was significantly associated with feeding difficulties, increased crying, and poorer sleep (Table 5).

### 3.4. Association Between Mothers’ Smartphone Use and SFS-Diagnosed Child Regulatory Disorder

A generalized linear model (GLM) was used for the interval-scaled subscores and total score of the SFS in data subset two. As a result, the mean values of the SFS subscores and total score are slightly higher in infants of mothers with frequent smartphone use than in infants of mothers with infrequent smartphone use (Table 6). Only the subscore for co-regulation showed an association, but the differences in mean values are not very relevant. The association found in the unadjusted analyses between frequent smartphone use by the mother and regulatory disorder in the child could not be confirmed in the adjusted model.

A binary logistic regression was performed including all potential confounding factors for the dichotomous variable of regulatory disorder of the child based on SFS sum score (Table 7). More frequent maternal smartphone use and increased maternal stress during smartphone use when child demands attention were both associated with a higher likelihood of regulatory disorder showing a small to moderate effect size. Conversely, the presence of a maternal psychiatric diagnosis as measured by the M.I.N.I. was associated with a slightly lower odds. No associations were observed for single parent status, maternal educational level, and maternal age.

## 4. Discussion

### 4.1. Summary of the Main Results

The aim of this study was to investigate the relationship between frequent parental smartphone use during interaction with their children and the occurrence of early regulatory disorders and to analyze the relevance of selected confounding factors. The study used two subsets from the SKKIPPI cohort: 5954 parent–child dyads and 972 mother–child dyads. Analyses of the first data subset revealed that in addition to the frequency of parental smartphone use, parental stress when interrupted during smartphone use was also consistently associated with less favorable outcomes across feeding, falling asleep, and crying domains. Single parent status and psychiatric history also emerged as associated with certain negative outcomes, while maternal age was generally associated with better outcomes.

In the second data subset, mothers with frequent smartphone use exhibited slightly higher scores on the SFS subscales and total score. The initially observed unadjusted association between frequent maternal smartphone use and regulatory disorders was not sustained after controlling for confounders in a generalized linear model, except for a small association in co-regulation. However, both frequent smartphone use and higher stress during use when the child demands attention were significantly associated with increased odds of the SFS-based diagnosis of early regulatory disorder. Maternal psychiatric diagnosis showed a weak negative association, and no relevant effects were found for education, age, or single parent status.

Overall, the findings suggest that maternal stress during smartphone use is more robustly associated with early regulatory difficulties than the frequency of smartphone use alone, indicating the importance of considering emotional and contextual factors in the analysis of technology use during parent–child interactions.

### 4.2. Comparison with Similar Studies

Our findings thus align with other research that already suggests that it is not smartphone use that is necessarily problematic, but rather the associated contextual factors or reactions [24,25]. Smartphone use does not necessarily mean that a parent is failing to respond to their child’s needs. The problem would instead be that the parent reacts inappropriately when disturbed or is not sensitive enough to the child’s needs.

In previous studies on this topic, the focus was primarily on the effects of increased technology use on caregivers [14,16,22] and only temporary changes in the behavior of infants and toddlers were described [19]. Nevertheless, the results of the still-face experiments modified for smartphone use, in which reduced exploratory behavior, reduced positive affect, and increased protest behavior in the children could be observed [19,20], suggest the possibility that parental technoference could be negatively associated with the bond between caregiver and child and the ability to learn self-regulation. An association was also found between frequent smartphone use reported by parents and more negative infant responses in the still-face experiment, which also supports the hypothesis of potential long-term effects and the differences observed in this study [19]. Even though these experiments only examined the child’s behavior at the moment of the experiment, the study design as an experiment with objective assessment of the reaction has clear methodological advantages compared with our cross-sectional study.

To date, however, there is a lack of studies on the association of technoference with changes in the behavior and self-regulation of infants in everyday life. The study by McDaniel and Radesky had a similar objective to the present study; however, the children were older, with an average age of 3 years, and the authors used different behavioral questionnaires [38]. They concluded that frequent parental use of digital technological devices classified as problematic predicted greater technoference in interactions with the child, with maternal technoference also predicting internalizing and externalizing behaviors of the child in reports from both parents. The authors thus demonstrated a link between technoference and problematic child behaviors [38]. Although these results are not directly transferable to infants, they also suggest the possibility of a correlation here. In some cases, very similar parameters were collected in the study design compared to this work, such as depression and comparable socio-demographic variables, and the results were adjusted for these. However, the difference between the significant results of McDaniel and Radesky and the ambiguous results of our study could be due to the more precise data collection on technology use using the Technology Device Interference Scale [39], differences in the study population, which included both the mother and the father of a child, and the analysis using Actor Partner Independence Modeling. As McDaniel and Radesky also referred to the subjective assessment of the parents in self-reported questionnaires, the risk of self-selection bias is also high here, as in our study. This applies in particular to the survey of the confounding factor depression with the Center for Epidemiologic Studies Depression Scale [40], whose diagnostic validity and scope are inferior to the M.I.N.I. interview used in this study.

Relatively unexpected, although plausible, was the strong correlation between parental perception of stress when using smartphones while interacting with the child, when the child demands attention, and the occurrence of regulatory disorders. Due to the screening nature of the online questionnaire on postnatal psychosocial stress in mothers, this item was only surveyed with one question in our study and was not explored further. In particular, we do not know how the parent reacted to the interruption of smartphone use, whether he or she reacted harshly or aggressively, or whether he or she was able to hide perceived stress from the child. A recent systematic review identified several studies in which “caregiver screen use” during interactions with their child was associated with poorer psychosocial outcomes for the child. However, the possible mechanisms underlying this were not investigated further [41]. In particular, the parent’s reaction when the child interrupts smartphone use by seeking attention was not examined or reported.

There is evidence of the link between maternal stress reaction and attachment problems [42]. A variety of postnatal psychosocial stressors are associated with an increased risk of excessive crying in children [43]. However, studies focus primarily on the significance of maternal mental disorders for the development of childhood regulatory disorders and a variety of other impairments to child health [44]. The extent of bidirectional causality is unclear: is parental stress promoted by the regulatory disorder, or is the regulatory disorder a consequence of parental stress [45]? Studies conducted during the coronavirus pandemic shift the focus away from maternal psychopathology to pure stress caused by external factors [46]. Mothers with higher stress levels had more difficulty putting their children to sleep, and infant crying and fussiness were also increased [47]. However, these studies focus on stress in general experienced by the mother or parents, rather than—as in our study—on the perception of stress when smartphone use is interrupted. Specific studies on the behavior of caregivers showing an elevated stress response when they are disturbed while using their smartphones are therefore useful because they would be relevant for the therapeutic approach to mother–child dyads.

### 4.3. Strengths and Limitations

One of the strengths of the present study was the relatively large study sample with a total of 5954 parent–child dyads in data subset one and 972 mother–child dyads in data subset two. The use of standardized and validated instruments such as the M.I.N.I. interview and the SFS questionnaire further underpin the reliability of the survey. Data subsets one and two used for the main analysis are not entirely independent; rather, the mother–child dyads in subset two were also included in subset one. However, the two subsets contain different types of data: subset one comprises self-reported information, whilst subset two comprises data from validated questionnaires and diagnostic interviews. The two different analyses yield similar results, which underscores the robustness of the findings. In addition, a sensitivity analysis conducted using subset one, which excludes the data records contained in subset two, confirms these findings. The strong association between a mother’s feeling of stress during smartphone use when the child demands attention and the child’s regulatory difficulties is thus shown to be a robust finding.

Nevertheless, there are several limitations that restrict the transferability and validity of the results. First, when looking at the response rate of 20% in the SKKIPPI cohort study, the influence of a response bias of the included participants is possible. For example, parents who have noticed problems such as regulatory difficulties in their infant could be more interested in participating in the study, while parents from multi-problem families or with severe (mental) illness could be prevented from taking the time to participate due to their stress, which could have significantly distorted the results collected here.

Second, other undiscovered and possibly very complex influencing factors are also likely, which could not be recorded and examined as part of this study. Additional confounding factors such as maternal personality characteristics, parenting style, infant temperament, social support, or overall screen time may also be relevant for parental smartphone use and infant regulation. One reason why we did not collect data on these additional factors was that, in order to keep the participation threshold as low as possible for the invited caregivers in screening step one of the SKKIPPI cohort study, not all aspects of the psychosocial stress of young parents in the postnatal period were asked in detail.

Third, the question about smartphone use behavior in the screening questionnaire was rather imprecise and was only answered based on the subjective assessment of the respondents, without being able to control or quantify this more precisely. Our dichotomization into two groups—(a) *frequently* and (b) *sometimes, rarely or not at all*—was, to a certain extent, artificial. An alternative dichotomization into (a) *frequently or sometimes* and (b) *rarely or not at all* might also result in the associations found being less pronounced. A more precise measurement of frequency of use and its inclusion as a continuously scaled variable is desirable for confirmatory analyses.

Fourth, only those parents who indicated psychosocial stress in the first screening stage were subjected to the further tests (SFS questionnaire and M.I.N.I. interview), so that ultimately the entire subpopulation in data subset two has a background of increased psychosocial stress.

Fifth, as the SFS questionnaire was completed by the mothers themselves, and no external observation of the interaction between mother and child took place, a bias due to different personal assessments and sensitivity is also possible at this point. In addition, because the same individual is evaluating both the exposure and the outcome, there may be a common-method bias. Stressed or psychologically burdened caregivers may be more prone to view their smartphone use and their baby’s behavior unfavorably.

Sixth, the threshold value of ≥1.85 in the sum score as a cut-off for the diagnosis of a regulatory disorder in the child may be critically questioned in this case, as the mean values are very close to this value for both infrequent and frequent smartphone use by the mother, and the association of the level of the score with the intensity of the clinical manifestation means that the severity of the symptoms may not differ as much in reality as the result suggests at first glance. The study describing the empirical basis of the SFS questionnaire also shows a broad distribution of clinically conspicuous and clinically inconspicuous infants, as well as a clear overlap of the groups around the score of 1.85 [36].

Seventh, a cross-sectional study design cannot generally prove causality, which is why further studies are necessary. For instance, caregivers who have babies with trouble eating, sleeping, or crying may experience more stress and use their smartphones more often as a coping mechanism. Even if the influence of response bias is difficult to prevent in population-based studies, a longitudinal study, which includes more extensive information on influencing factors, temporal ordering, and which quantifies the use of technological devices more precisely would be desirable for future research.

## 5. Conclusions

The present study indicates that frequent smartphone use by parents while interacting with their infant is associated with less favorable outcomes across feeding, falling asleep, and crying domains. In addition, maternal stress during smartphone use when the child demands attention turned out to be at least as strongly associated with the child’s regulatory problems. Although the results should be interpreted with caution due to the potential limitations of our study, they are highly relevant for further research into parental technoference in childhood. In particular, further research is needed to determine the long-term consequences of this on early childhood self-regulation, attachment, and possible late effects on development and the child’s mental health. The caregivers’ observed experience of stress also suggests the importance of future studies of parental perspective in terms of the effects of intensive smartphone use or even addiction on the relationship with their child.

This study suggests that when counseling or treating parent–child dyads with early regulatory disorders, parents’ smartphone use should be explored in terms of behavior toward the child during smartphone use, including signs of typical addictive behavior. Early childhood education should provide parents with information on guideline-aligned and age-appropriate media consumption and age-appropriate regulation strategies [48]. Smartphone use alone should not be interpreted as harmful per se, but context, content, and behavior when using smartphones should be given more detailed attention.

## Figures and Tables

**Table 1:** Variables used in subsets 1 and 2 from the SKKIPPI cohort study data [26,28].

	Data Subset 1 (N = 5954)	Data Subset 2 (n = 972)
Screening step 1 (online questionnaire)	− Sociodemographic variables − Caregiver’s history of mental health problems − Caregiver’s smartphone use − Parent-reported regulatory problems of the child − Caregiver’s perceived stress during smartphone use when child demands attention	− Sociodemographic variables − Mother’s history of mental health problems − Mother’s smartphone use − Parent-reported regulatory problems of the child − Mother’s perceived stress during smartphone use when child demands attention
Screening step 2 (telephone diagnostic interview M.I.N.I. and validated paper & pencil questionnaires)	-	− M.I.N.I. diagnosis − SFS questionnaire − Regulatory disorder based on SFS

**Table 2:** Basic sociodemographic data of the study population of 5954 parent–child dyads from data subset one, stratified into groups with frequent and infrequent parental smartphone use, represented by mean value (standard deviation SD) and range or by absolute frequencies (relative frequencies).

	Total Group (N = 5954) ^1^	Frequent Smartphone Use (n = 218, 3.7%)	Infrequent Smartphone Use (n = 5707, 96.3%)
Age, years (SD) range	34.1 (4.7) 18–63	33.3 (4.7) 23–49	34.2 (4.7) 18–63
Caregiver, n (%) Mother	4984 (83.7)	189 (86.7)	4783 (83.8)
Father	963 (16.2)	29 (13.3)	922 (16.2)
Caregiver not specified	7 (0.1)	0	2 (0.0)
Education level, n (%) ISCED I	31 (0.5)	0 (0)	31 (0.5)
ISCED II	822 (13.8)	26 (11.9)	796 (13.9)
ISCED III	4995 (83.9)	189 (86.7)	4806 (84.2)
Other	34 (0.6)	1 (0.5)	33 (0.6)
Education not specified	72 (1.2)	2 (0.9)	41 (0.7)
Single parent, n (%) No	5584 (93.8)	202 (92.7)	5381 (94.3)
Yes	297 (5.0)	14 (6.4)	283 (5.0)
Status not specified	73 (1.2)	2 (0.9)	43 (0.8)
Index ^2^ child is first child, n (%) No	2459 (41.3)	95 (43.6)	2358 (41.3)
Yes	3476 (58.4)	123 (56.4)	3349 (58.7)
Child’s status not specified	19 (0.3)	0 (0)	0 (0)
Receipt of state benefits, n (%) No	5141 (86.3)	184 (84.4)	4956 (86.8)
Yes	738 (12.4)	32 (14.7)	706 (12.4)
Benefits not specified	75 (1.3)	2 (0.9)	45 (0.8)
Lifetime experience of a psychiatric disorder (self-reported), n (%) None	4713 (79.2)	171 (78.4)	4542 (79.6)
At least one	1165 (19.6)	44 (20.3)	1121 (19.7)
Experience not specified	76 (1.3)	3 (1.4)	44 (0.8)
Feeling stress during smartphone use when child demands attention, n (%) No	4981 (83.7)	149 (68.3)	4832 (84.7)
Yes	938 (15.8)	69 (31.7)	869 (15.2)
Feeling not specified	35 (0.6)	0 (0)	6 (0.1)
Parent-reported regulatory problems of the child, n (%)			
Feeding child is easy Yes	5656 (95.0)	198 (90.8)	5456 (95.6)
No	264 (4.4)	20 (9.2)	244 (4.3)
Not specified	34 (0.6)	0 (0)	7 (0.1)
Child falls easily asleep, n (%) Yes	362 (6.1)	16 (7.3)	346 (6.1)
No	5561 (93.4)	202 (92.7)	5359 (93.9)
Not specified	29 (0.5)	0 (0)	2 (0.0)
Child cries excessively, n (%) Yes	1662 (27.9)	66 (30.3)	1596 (28.0)
No	4264 (71.6)	152 (69.7)	4111 (72.0)
Not specified	28 (0.5)	0 (0)	0 (0.0)

^1^ Missing information on smartphone use for 29 participants. ^2^ The index child was the child who was registered as a newborn at the residents’ registration office and was recruited together with its mother as part of the representative population sample for the SKKIPPI study. The mother may already have other children.

**Table 3:** Basic sociodemographic data, mothers’ psychiatric disorders, and SFS scores of the study population of 972 mother–child dyads from data subset two, stratified into groups with mothers’ frequent and infrequent smartphone use, represented by mean value (standard deviation SD) and range or by absolute frequencies (relative frequencies).

	Total Group (N = 972)	Frequent Smartphone Use (n = 56, 5.8%)	Infrequent Smartphone Use (n = 916, 94.2%)
Age, in years (SD) range	34.4 (4.7) 20–50	33.2 (5.4) 24–49	34.4 (4.7) 20–50
Education level, n (%) ISCED I	5 (0.5)	0 (0)	5 (0.5)
ISCED II	141 (14.5)	4 (7.1)	137 (15.0)
ISCED III	815 (83.8)	52 (92.9)	763 (83.3)
Other	11 (1.1)	0 (0)	11 (1.2)
Single parent, n (%) No	865 (89.0)	48 (85.7)	817 (89.2)
Yes	107 (11.0)	8 (14.3)	99 (10.8)
Index child is first child, n (%) No	420 (43.2)	23 (41.1)	397 (43.3)
Yes	552 (56.8)	33 (58.9)	519 (56.7)
Receipt of state benefits, n (%) No	789 (81.2)	46 (82.1)	743 (81.1)
Yes	181 (18.6)	10 (17.9)	171 (18.7)
Benefits not specified	2 (0.2)	0 (0)	2 (0.2)
Psychiatric disorder (according to M.I.N.I), n (%)			
None	705 (72.5)	41 (73.2)	664 (72.5)
At least one	267 (27.5)	15 (26.8)	252 (27.5)
Feeling stress during smartphone use when child demands attention, n (%) No	732 (75.3)	33 (58.9)	699 (76.3)
Yes	240 (24.7)	23 (41.1)	217 (23.7)
SFS sum score	1.83 (0.3)	1.91 (0.3)	1.82 (0.3)
SFS subscore “crying, whining, sleeping”	1.75 (0.4)	1.79 (0.3)	1.75 (0.4)
SFS subscore “co-regulation”	2.40 (0.6)	2.56 (0.6)	2.38 (0.6)
SFS subscore “feeding”	1.45 (0.4)	1.52 (0.4)	1.44 (0.4)
SFS-based diagnosis of regulatory disorder (n datasets in analysis) (n, %)	834	52	782
No	477 (57.2)	20 (38.5)	457 (58.4)
Yes	357 (42.8)	32 (61.5)	325 (41.6)

**Table 4:** Association of parental smartphone use, perceived stress during smartphone use when the child demands attention, parents’ demographic characteristics, and parent-reported lifetime psychiatric experience (n = 5954 from data subset one) with parent-reported regulatory disorders of the child.

	Odds Ratio (95% CI)	*p*-Value
**Parent-reported child feeding easy**		
Parent’s frequent smartphone use (vs. infrequent use)	0.51 (0.31; 0.83)	0.007
Parent’s elevated stress level when disturbed during smartphone use (vs. no elevated stress)	0.56 (0.42; 0.75)	<0.001
Parent is single parent (vs. not single)	1.50 (0.78; 2.88)	0.226
Parent’s educational level is high (vs. medium or low)	1.20 (0.87; 1.66)	0.272
Parent’s age (per year)	1.00 (0.97; 1.03)	0.859
Parent’s psychiatric experience (yes vs. no)	0.77 (0.58; 1.04)	0.088
**Parent-reported child falling asleep easily**		
Parent’s frequent smartphone use (vs. infrequent use)	0.93 (0.69; 1.26)	0.650
Parent’s elevated stress level when disturbed during smartphone use (vs. no elevated stress)	0.85 (0.73; 0.99)	0.036
Parent is single parent (vs. not single)	0.75 (0.58; 0.96)	0.022
Parent’s educational level is high (vs. medium or low)	0.88 (0.76; 1.03)	0.120
Parent’s age (per year)	1.03 (1.01; 1.04)	<0.001
Parent’s psychiatric experience (yes vs. no)	0.79 (0.68; 0.91)	<0.001
**Parent-reported excessive crying of child**		
Parent’s frequent smartphone use (vs. infrequent use)	1.08 (0.63; 1.86)	0.773
Parent’s elevated stress level when disturbed during smartphone use (vs. no elevated stress)	1.37 (1.04; 1.81)	0.024
Parent is single parent (vs. not single)	1.54 (1.02; 2.33)	0.039
Parent’s educational level is high (vs. medium or low)	0.82 (0.62; 1.07)	0.134
Parent’s age (per year)	0.97 (0.95; 0.99)	0.007
Parent’s psychiatric experience (yes vs. no)	1.28 (0.99; 1.66)	0.058

**Table 5:** Association of parental smartphone use, perceived stress during smartphone use when the child demands attention, parents’ demographic characteristics, and self-reported lifetime psychiatric experience (n = 4982 from data subset one, records of the mothers included in subset two were excluded) with parent-reported regulation disorders of the child.

	Odds Ratio (95% CI)	*p*-Value
**Parent-reported child feeding easy**		
Parent’s frequent smartphone use (vs. infrequent use)	0.48 (0.26; 0.89)	0.020
Parent’s elevated stress level when disturbed during smartphone use (vs. no elevated stress)	0.52 (0.36; 0.75)	<0.001
Parent is single parent (vs. not single)	1.87 (0.74; 4.75)	0.189
Parent’s educational level is high (vs. medium or low)	1.27 (0.86; 1.89)	0.227
Parent’s age (per year)	0.98 (0.95; 1.01)	0.157
Parent’s psychiatric experience (yes vs. no)	0.95 (0.62; 1.46)	0.826
Child in the study is parent’s first child (vs. subsequent child)	0.63 (0.45; 0.88)	0.007
Parent receives state benefits (vs. no state benefits)	0.60 (0.39; 0.94)	0.025
**Parent-reported falling asleep easily**		
Parent’s frequent smartphone use (vs. infrequent use)	1.01 (0.71; 1.45)	0.943
Parent’s elevated stress level when disturbed during smartphone use (vs. no elevated stress)	0.78 (0.66; 0.94)	0.007
Parent is single parent (vs. not single)	0.77 (0.56; 1.06)	0.112
Parent’s educational level is high (vs. medium or low)	0.91 (0.76; 1.09)	0.310
Parent’s age (per year)	1.01 (1.00; 1.03)	0.165
Parent’s psychiatric experience (yes vs. no)	0.93 (0.77; 1.12)	0.440
Child in the study is parent’s first child (vs. subsequent child)	0.63 (0.54; 0.72)	<0.001
Parent receives state benefits (vs. no state benefits)	0.83 (0.67; 1.02)	0.072
**Parent-reported excessive crying**		
Parent’s frequent smartphone use (vs. infrequent use)	1.07 (0.54; 2.14)	0.845
Parent’s elevated stress level when disturbed during smartphone use (vs. no elevated stress)	1.51 (1.07; 2.13)	0.018
Parent is single parent (vs. not single)	1.26 (0.71; 2.22)	0.434
Parent’s educational level is high (vs. medium or low)	0.82 (0.59; 1.14)	0.236
Parent’s age (per year)	0.97 (0.95; 1.00)	0.082
Parent’s psychiatric experience (yes vs. no)	0.74 (0.49; 1.14)	0.169
Child in the study is parent’s first child (vs. subsequent child)	1.23 (0.93; 1.64)	0.155
Parent receives state benefits (vs. no state benefits)	1.93 (1.35; 2.78)	<0.001

**Table 6:** Infant regulatory disorders according to SFS in the study population (n = 972 from data subset two in analysis), stratified into groups with frequent and infrequent smartphone use, adjusted for the presence of at least one psychiatric diagnosis according to M.I.N.I. in the mother, educational level, age, and single parent status of the mother, and subjective feeling of stress during smartphone use when child demands attention.

	Frequent Smartphone Use (Mean, 95% CI)	Infrequent Smartphone Use (Mean, 95% CI)	*p*-Value
SFS sum score (n in analysis)	52	782	
	1.91 (1.82; 2.00)	1.82 (1.80; 1.84)	0.052
SFS subscore “crying, whining, sleeping” (n in analysis)	52	782	
	1.79 (1.69; 1.90)	1.75 (1.72; 1.77)	0.388
SFS subscore “co-regulation” (n in analysis)	52	778	
	2.56 (2.40; 2.73)	2.38 (2.34; 2.43)	0.043
SFS subscore “feeding” (n in analysis)	52	777	
	1.52 (1.42; 1.63)	1.44 (1.42; 1.47)	0.148

**Table 7:** Association of smartphone use, presence of at least one psychiatric diagnosis according to M.I.N.I. in the mother, educational level, age, and single parent status of the mother, and subjective feeling of stress during smartphone use when the child demands attention, and diagnosis of regulatory disorder, defined by the cut-off at sum score ≥ 1.85 in the SFS (Questionnaire for Crying, Feeding and Sleeping) (n = 834 from data subset two).

	Odds Ratio (95% CI)	*p*-Value
**Diagnosis of regulatory disorder**		
Mother’s frequent smartphone use (vs. infrequent use)	2.00 (1.11; 3.60)	0.021
Mother’s elevated stress level when disturbed during smartphone use (vs. no elevated stress)	1.87 (1.35; 2.58)	<0.001
Mother is single parent (vs. not single)	1.02 (0.65; 1.59)	0.947
Mother’s educational level high (vs. medium or low)	1.07 (0.75; 1.54)	0.702
Mother’s psychiatric diagnosis (diagnosed by M.I.N.I. vs. no diagnosis)	0.97 (0.94; 0.99)	0.018
Mother’s age (per year)	1.21 (0.89; 1.65)	0.232

## Data Availability

The data presented in this study are available on request from the corresponding author. The data are not publicly available due to privacy and ethical reasons.
